# *I-BLEND*, a campus-scale commercial and residential buildings electrical energy dataset

**DOI:** 10.1038/sdata.2019.15

**Published:** 2019-02-19

**Authors:** Haroon Rashid, Pushpendra Singh, Amarjeet Singh

**Affiliations:** 1 Computer Science and Engineering, OKhla Phase III, IIIT Delhi, New Delhi, 110020 India

**Keywords:** Energy infrastructure, Energy and behaviour, Energy efficiency, Energy modelling, Power distribution

## Abstract

Efficient energy consumption at the building level is vital for sustainability. Providing energy efficient systems and solutions requires an understanding of how energy gets consumed. However, there is a general lack of large-scale open datasets about the energy consumption of buildings, which hinders the research. The recent emergence of smart energy meters makes it possible to collect such data, which can then be used for analysis. In this paper, we release *I-BLEND*, 52 months of electrical energy dataset at a one-minute sampling rate from commercial and residential buildings of an academic institute campus in an emerging economy, India. Also, we provide occupancy datasets at a 10-minute sampling rate for each of the campus buildings. To the best of our knowledge, this is the first such dataset from India. Public availability of such fine-granular data will allow users to perform different research tasks such as analyzing the impact of weather or occupancy schedule on energy consumption, detecting anomalies, and developing algorithms for predictive maintenance.

## Background & Summary

Energy sustainability is one of the top ten problems of humanity as identified by Richard E. Smalley^1^. Buildings consume a significant portion (41%) of available electrical energy^2^. The energy efficiency of buildings is one of the important ways of handling energy sustainability, and the process includes identifying ways to reduce energy consumption and proposing energy efficient solutions^3^.

The energy efficiency of buildings starts with an understanding of energy consumption, i.e., how buildings consume energy over a period under different conditions (e.g., seasons, occupancy, etc.). The increasing deployment of smart meters can be seen as an enabler of energy efficiency. In addition to billing purposes, these meters allow the logging of different energy parameters (voltage, power factor, and others) as high as 1 Hertz rate and can provide appliance-level consumption details through an energy disaggregation approach^4^. India has 120 million registered consumers (residential, commercial, industrial, and agricultural). Till now, 5.2 million smart meters have been installed; another 35 million are expected to be installed by 2019^5,6^.

Smart meters measure different energy parameters. Data collection from such meters is still a painful process due to the unreliable nature of data collection hardware, communication issues, and the continuous monitoring of the data collection setup. Due to these challenges, it is often difficult to make large datasets ready for public release, especially from developing regions where technology is still in its infancy. Some of the major publicly available energy datasets, as shown in Table 1, include REDD, Dataport, ECO, and UK-DALE among others. These datasets span from days to years with data collected from a few to 100 s of buildings. From India, only one dataset from a single household is available. It spans a period of 73 days^7^.

In this paper, we release our dataset from India, namely the Indian BuiLdings Energy coNsumption Dataset (*I-BLEND* ) (Data Citation 1). *I-BLEND* is a 52-month energy dataset from seven buildings (commercial and residential) of an academic institute campus. This dataset contains five different parameters-voltage, current, power, frequency, and power factor-at a sampling frequency of one minute.

The unique characteristics which make *I-BLEND* interesting include: (*i*) different environmental factors-voltage fluctuations due to poor infrastructure and supply deficit, power failure, and poor network connectivity^7^, (*ii*) a mix of commercial and residential buildings on an academic campus (both types of buildings vary in terms of operational hours and energy consumption patterns, and hence offer diverse research opportunities), and (*iii*) the long duration of the dataset makes it a good fit for deep learning and neural network applications^8^. The *I-BLEND* dataset also fills the gap from emerging economies.

With *I-BLEND*, we release other supplementary data which includes occupancy, institute calendar, building architecture details, and four months of local weather (temperature, humidity) (Data Citation 1). The weather parameters (temperature, humidity, wind speed, and wind direction) of a nearby weather station are already publicly available at 30 min intervals from a free weather service, Weather Underground (WU)^9^. The occupancy dataset is at 10-minute resolution for all seven buildings.

Researchers and the academic community may use the dataset, including but not limited to, in the following ways:

1. Analyze the change in energy consumption over a period.
2. Evaluate demand side management algorithms, such as Demand Response^10^.
3. Understand the impact of climate and occupancy on consumption.
4. Evaluate various applications, such as anomaly detection, prediction, identifying correlations in the consumption of campus buildings.
5. Understand the impact of various energy parameters (current, voltage, and others) on one another.

## Methods

### Building characteristics

Indraprastha Institute of Information Technology Delhi (IIIT-D), spread over 25 acres, is an autonomous research institute in Delhi, India. The campus of the institute started in 2012 with newly constructed buildings. It has seven buildings, namely, Academic, Lecture, Library, Facilities, Dining, Boys dormitory, and Girls dormitory. The energy demand of all buildings are met by electricity received from the state electricity board; diesel generators are used in the events of power cuts. Table 2 provides an overview of these buildings. The average minimum temperature in Delhi during winters is around 8° Celsius, and average maximum temperature during summers reaches to around 42° Celsius. All buildings on campus, except Facilities building, are connected to a centralized Heating, Ventilation, and air Conditioning (HVAC) system. Therefore, the total energy consumed by these buildings (excluding facilities) includes the energy consumed by HVAC components such as air handler units (AHU). Almost every building is used for a different purpose, so electrical loads used and operational timings vary for each of them. The following description provides an overview of the characteristics of each building.

#### Academic Building

This building consists of faculty offices, server room (hosting most of the computer servers on campus), and research labs used by research scholars. The electrical loads in this building include computers, fans, lights, AHUs, and two lifts. Most faculty offices remain open from 0800 till 1800 hours, whereas research scholars do not follow a particular schedule, and hence, the building remains occupied with limited numbers during night hours and on Saturdays and Sundays as well.

#### Lecture Building

This building consists of nine classrooms. Typically, classes finish on weekdays by 1730 hours, but on some special events (such as “hack nights”), with an average frequency of one per month, a few classes remain occupied till midnight. Electrical loads in this building include lights, fans, and fan coil units of HVAC.

#### Facilities Building

This building consists of five administrative offices and an electrical panel room. Major electrical loads in this building include lights, fans, and seven window ACs.

#### Library Building

This building consists of an open area, library, and computer labs. The open area remains open 24 × 7 for reading; the library remains open from 0830 until midnight. Electrical loads in this building include lights, fans, two lifts, AHUs, and three computer labs, consisting of around 150 computers in total, for courses.

#### Dining Building

This building consists of a cafeteria which remains open 24 × 7, a dining floor, computer labs, and a floor with a gym and table tennis rooms. Electrical loads in this building include lights, fans, a lift, AHUs, computers, and gym equipment (three treadmills).

#### Dormitory Buildings

There are separate dormitories for boys and girls. Each dormitory has single and shared (double, triple) rooms and four lifts. Electrical loads in these buildings include lights, fans, AHUs, and high wall units. The HVAC unit remains operational in these buildings during night hours only. Dormitories have both mains and UPS power supply. Single rooms have one tube light, one fan (on UPS), two plug points (one on mains and another on UPS), one study light (on UPS ), and an AC high wall unit (on mains). In the case of the shared rooms, all loads or plug points are multiplied by 2 (or 3) according to sharing type. Students are allowed to use electricity for laptops, desktops, and mobile phones only.

Table 3 shows the number of students staying in boys’ and girls’ dormitories during three different sessions for three years. Months January to April and August to November represent two academic semesters and months December and May to July represent winter and summer vacations respectively. During vacations, only graduate students remain on campus. During academic semesters, the number of boys staying in dormitories are approximately double as compared to the number of girls.

### Metering and data-collection setup

Firstly, we explain the metering part, and then we explain the data-collection, storage, and retrieval part.

#### Metering at aggregate building level

We use standard panel meters (Schneider EM6400^11^) designed for industrial and commercial installations to measure the different electrical parameters. Each unit costs approximately $150. They can measure, display, and communicate approximately 25 different electrical parameters using Modbus protocol. They are three-phase meters and receive input voltage signals via three potential transformers (PTs) and current signals via three current transformers (CTs). Using these voltage and current signals, other electrical parameters are computed internally using various formulae mentioned in the User Manual^11^. They store these parameters temporarily in registers, and these registers are updated every few seconds (we experimentally observed the update interval to be two seconds). In accordance with IEC 62053-21 standards, they have an accuracy class of 1.0, meaning that measurement error can be up to 1 percent. They are pre-calibrated meters, and the manufacturer does not recommend any subsequent recalibration. Over a duration of 4.5 years, we did not find any case of meter breakdown.

**Figure 1:**
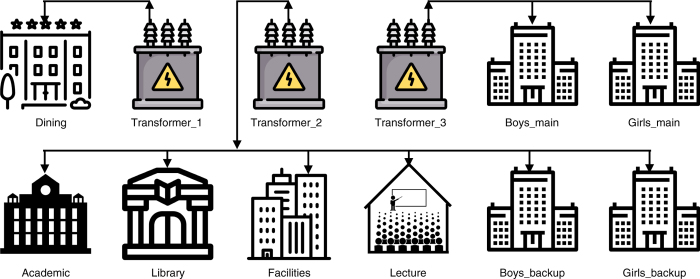
Flow diagram of transformers and building meters. Transformer_1 and Transformer_2 have several auxiliary loads that are not released in the paper.

Each building on campus has a separate EM6400 meter installed on building mains. The institute has three transformers, which supply energy to different buildings on campus, and each transformer has a meter installed. We label these transformers as Transformer_1, Transformer_2, and Transformer_3. The mains and UPS consumption in dormitories are measured with separate meters, and accordingly, meter names are suffixed with the words “mains” or “backup” (e.g., Boys_main, Boys_backup). Figure 1 shows a flow diagram between the transformers and the different buildings on campus.

#### Data collection from building level meters

**Figure 2:**
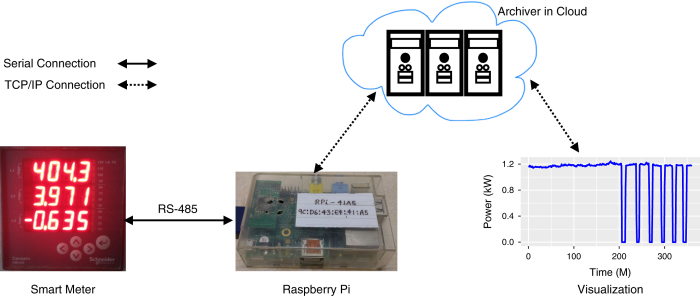
Data collection and visualization setup.

We use an open source Simple Measuring and Actuation Profile (sMAP) platform to collect, store, and retrieve the data^12^. Figure 2 provides an overview of the entire setup. The EM6400 meters connected to the building’s main supply measure different parameters such as voltage, current, power, frequency, and power factor. These meters are then connected to Raspberry Pi modules via RS-485 serial transmission standard, and the measured parameters are collected from meters to Raspberry Pi through a Modbus communication protocol. A self-designed USB-RS485 converter is used to read data from meters into the sMAP application. The sMAP application, running in Raspberry Pi, polls these meters at a frequency of 30 seconds and temporarily stores the response data (parameter values) on the memory card of the Raspberry Pi. Raspberry Pis are connected to the reliable Internet via an Ethernet interface, so all Pis remain time synchronized. In real-time, sMAP components on the same Raspberry Pi format each received parameter values from a smart meter by assigning a unique identifier and attaching the required metadata. The sMAP modules on Pi publish these time-series parameters to another sMAP module in Cloud, called as *Archiver*, where this data is stored in a *readingdb* database^13^. The sMAP Archiver provides API for both visualizing and fetching the data.

### Occupancy data

With the ubiquity of wireless devices (laptops, smartphones, etc.), Wi-Fi infrastructure is increasingly being used for indoor localization, buildings occupancy monitoring, and footfall measurement^14–20^; and it has been found comparable in accuracy to other occupancy monitoring techniques. IIIT-Delhi, being a newly constructed academic campus, has a rich deployment of wireless Cisco Access Points (series 1100, 1600, 1800, 2800, 3700, and 1500) across the campus and has seamless wireless Internet access. Each access point covers a radius of around 15 meters. On top of the Wi-Fi infrastructure, we have built a separate system^18,19^, which collects SNMP (Simple Network Management Protocol) traps of all the buildings on campus. It has been operational since February 16, 2014. A trap is a data packet generated by a wireless Access Point whenever a client (laptop, mobile phone, etc) connects or disconnects with the access point. Each trap distinguishes itself from the remaining traps as each trap is associated with unique information consisting of a client_id, an access point_id, the trap type, a timestamp, etc. The access point_id is created such that it acts as a building identifier, e.g., ACB3FAP2 refers to the second access point that is installed on the third floor of the academic building. All traps are forwarded to a central server, which creates a log of all received traps. Analysis of these logs provides various details, such as how long a client was connected to a specific access point or how many clients are connected to an access point. We use these Wi-Fi logs to extract the occupancy details for each building on the campus. We first separate traps into different groups using a building identifier and later compute the occupancy count for each building separately using Box 1. The occupancy system is explained in detail in^18,19^.

By default, the occupancy dataset is at a one-minute resolution, and for this paper, we downsampled it to a 10-minute resolution by taking the maximum value of each 10-minute consecutive window. Downsampling does not reduce the occupancy accuracy significantly as the average difference found between a maximum and a minimum values of a window is eight, i.e., downsampling at max reduces the occupancy accuracy by eight occupants. The supplied occupancy data is from February 16, 2014, to November 3, 2017 (almost four years).

### Weather data

Weather data is publicly available from a free weather service, namely, Weather Underground (WU)^9^. The weather station is deployed and maintained by the Indian Metrological department, and the data is available through WU API. This weather station records about 13 weather parameters at a nearby location, IGIA Airport, New Delhi, which has the coordinates: 28.5667° latitude, 77.1167° longitude, and 237 meters elevation. The coordinates of IIIT-Delhi campus buildings are 28.5463° latitude, 77.2732° longitude, and 226 meters elevation. The distance between the airport weather station and IIIT-Delhi campus buildings is approximately 14.8 Km. The half-hourly weather data of the station was downloaded using WU API. We have downloaded temperature, humidity, wind speed, and wind direction data for the same duration as the power consumption data with our script at GitHub^21^. The exact position and the make and model of the measuring instruments at the IGIA Airport was not revealed to us by the Indian Meteorological department.

To understand the difference in weather at IIIT-Delhi compared to the airport, we measured the outdoor temperature and humidity at IIIT-Delhi campus with an off-the-shelf Elitech RC-4HC^22^ temperature and humidity data logger, costing around $120, continuously for four months. The data logger was fixed on an outside wall facing south but was shielded from direct sunlight. It has a temperature accuracy and resolution of±0.5°C and 0.1°C respectively, and humidity accuracy of ±3%RH. The Pearson Correlation coefficient of the temperatures at the two sites (IIIT-Delhi and IGIA Airport) is 0.96, and that of humidity is 0.90. This suggests that temperatures and humidity at the two sites vary in the same pattern.

**Figure 3:**
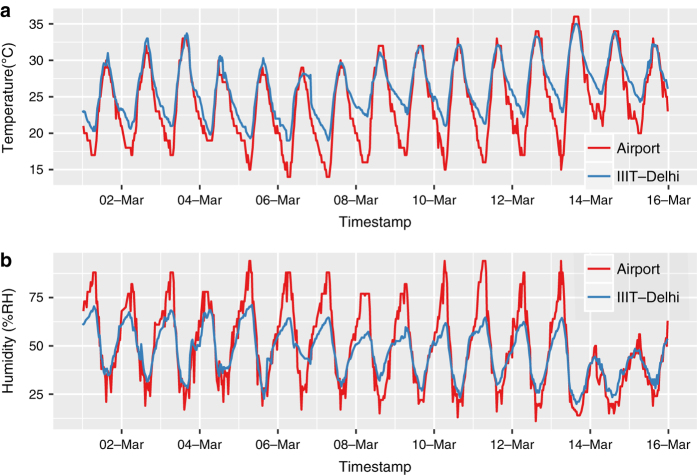
Half-hourly measurements at IGIA Airport and IIIT-Delhi campus from March 1, 2018 until March 15, 2018: (**a**) temperature, (**b**) humidity.

Figure 3(a) shows the variation in temperature at both sites for two continuous weeks, and Table 4 shows mean temperature and standard deviation at both sites during daytime (8 AM - 6 PM) and nighttime hours separately. When comparing these sites, daytime temperatures did not differ, but during night hours, a mean difference of 1.81 Celsius was found. The airport area is a non-residential open area, while IIIT-Delhi (Okhla, Phase III) is a residential-cum-industrial area. As a result, nighttime temperatures are relatively lower at the airport station when compared to the IIIT-Delhi station. Similarly, Fig. 3b shows the variation in humidity at both sites. A mean difference of 4.82 and 1.58 is found in humidity among the two sites during the day and night hours respectively. The insignificant difference in weather between the two sites suggests that IGIA weather data can be used to study the impact of weather variables on IIIT-Delhi buildings’ energy consumption.

### Calendar

The Institute has a calendar which shows working, non-working, and the semester days. We encode all this information in three column CSV file where the first column contains dates, the second column shows whether the day was working or not, and the third column shows whether the day was classified as high or low activity period. High activity period corresponds to days when the academic semester was going on, and students were in campus dormitories. Semester breaks, summer and winter breaks, and festival breaks of several consecutive days during the semesters were considered as low activity period. This information would be helpful for energy forecasting applications.

### Code availability

The sMAP code used to collect and store data is publicly available at GitHub^23^. The scripts used to process the data and plot results for this paper are publicly available at *I-BLEND* GitHub^24^.

## Data Records

Energy data from each building is stored in the form of CSVs (Data Citation 1). Each CSV contains six columns corresponding to Unix timestamp, power (watts), current, voltage, frequency, and power factor. The Unix timestamp measures the number of seconds since January 1, 1970. This timestamp can be converted to a human-readable format with any software by specifying the timezone as “Asia/Kolkata”, which has an offset of +5:30 hours from UTC. All readings are aligned at a one-minute sampling rate. A computer set in a different timezone might convert Unix timestamps to human readable format incorrectly, so we provide Python^25^ and R^26^ scripts to read and convert timestamps appropriately.

### Data Cleaning

The smart meters installed at the campus log data every 30 s. We downsampled data to a uniform one minute rate because of the following reasons: (i) readings from different meters were not time synchronized with one another, so for better comparison we aligned them at uniform one-minute durations, and (ii) the dataset is not targeted for energy disaggregation research, so the sampling frequency of 1 min is good enough for remaining applications, such as forecasting and benchmarking.

**Figure 4:**
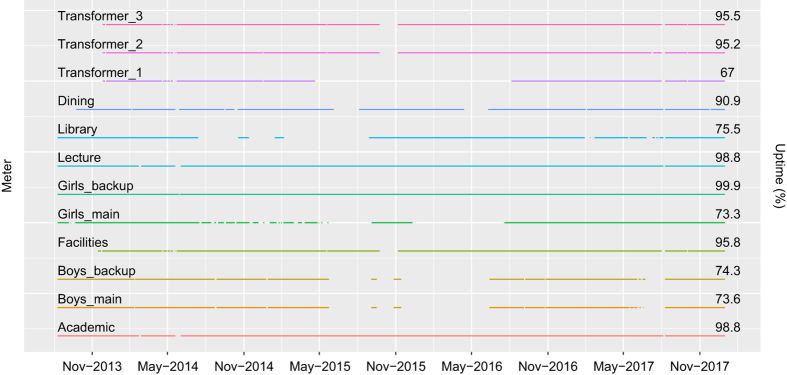
Gaps in the lines represent days on which more than a quarter of the meter readings are missing. Uptime represents the percentage of days for which more than a quarter of the meter readings are present.

Data logging for the academic building, the lecture building, dormitories, and the library building started from August 10, 2013. And for the facilities building and transformers, it started from November 15 and 26, 2013 respectively. For this paper, we have considered data until December 31, 2017. Gaps in Figure 4 show the different days in which more than a quarter of the readings ($>\frac{1440}{4}$) from a particular meter were missing. The secondary y-axis of the plot shows the number of days in percentage when the meter has more than a quarter of readings. We denote this with the uptime metric. This plot shows that meters, namely, Academic, Girls_backup, and Lecture, have the least missing data. Meters showing similar gap patterns are controlled by the same Raspberry Pi. Reasons for missing data include faulty power supply or corrupted memory card of Raspberry Pi. Running Little’s test^27^ for Missing Completely at Random (MCAR), we got a *p* value of 0.51, suggesting that all missing instances are MCAR, and hence, no hidden factor controls the pattern of such missing observations.

## Technical Validation

In this section, first, we will show the technical validation of the energy data and later of the occupancy data.

### 0.1 Energy data

**Figure 5:**
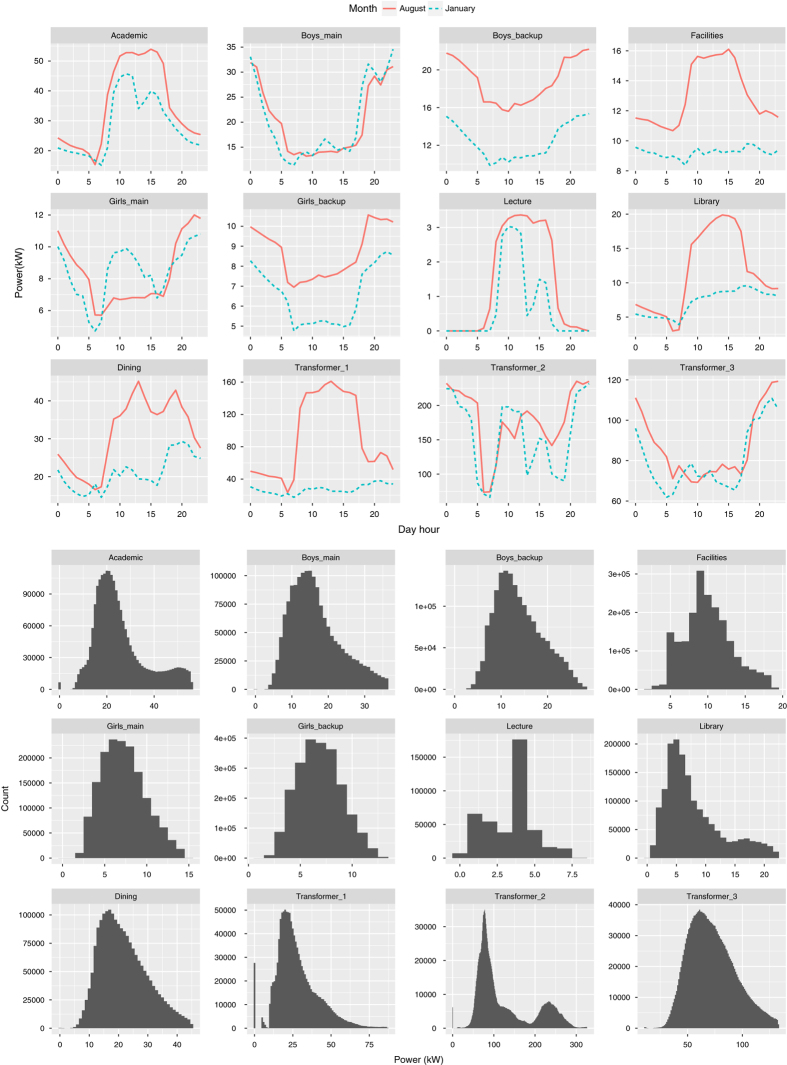
Top: Average power consumption across the same hours on different days of Aug. 2016 & Jan. 2017. (Months with different seasonality pattern). Bottom: Histograms of the power consumption of different meters from August 2013 until December 2017 when the histogram bin width is 1 kW.

Figure 5 (top) shows the average power consumption at different daytime hours in August 2016 and January 2017. These months differ in terms of academic semesters, and seasonality and hence represent summer and winter consumption patterns. The academic building remains occupied mostly between 0800 and 1800 hours, so the consumption is highest in this time range as compared to the remaining daytime hours. The lecture building follows a strict daily operation schedule because most classes finish by 1730 hours with the exception of when some extra classes happen. Dormitories consume more power during the nighttime than during the daytime since all students remain inside their rooms. Consumption in facilities building remains constant during the night and day in January 2017 as it is isolated from the centralized HVAC system. While the higher consumption during daytime hours in August 2016 is due to window ACs. The Dining building has some fixed usage due to the gym and labs; apart from this, its consumption increases at lunch and dinner time.

Figure 5 (bottom) shows histograms of the power consumption of all buildings and transformers from August 2013 to December 2017. These plots indicate that academic building follows a bimodal distribution having 20 and 50 kW frequently occurring loads. 50 kW represents working hours consumption, and 20 kW represents night hours consumption. Boys’ and girls’ dormitories have 31.5 kW (17.5 of main and 14 of backup) and 13.8 kW (7 of main and 6.8 of backup) frequently occurring loads, respectively. Facilities, Lecture, Library, and Dining have frequently occurring loads of around 10, 2, 10 and 20 kW, respectively.

**Figure 6:**
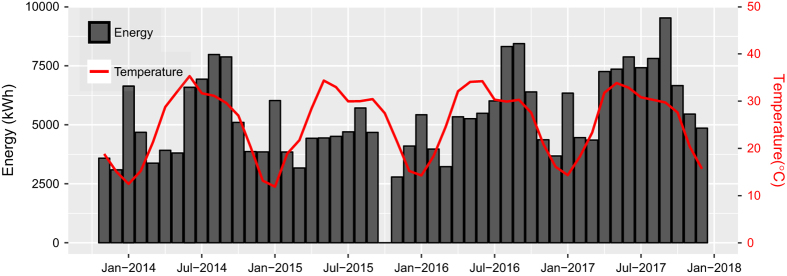
Monthly average energy consumption and temperature from November 26, 2013, until December 2017.

Figure 6 shows the monthly average energy consumption of the campus (sum of transformers 1, 2, and 3) and temperature from 2013 until 2017. Note in Fig. 4, that the meter connected to Transformer_1 did not log data from May 2015 until August 2016, and meters of the remaining two transformers did not log data for October 2015. As a result, in Fig. 6, the increase in energy consumption during summer 2015 is not clear as found in the remaining years of the figure. The higher consumption during summers and January is due to cooling and heating loads, respectively.

### 0.2 Occupancy data

**Figure 7:**
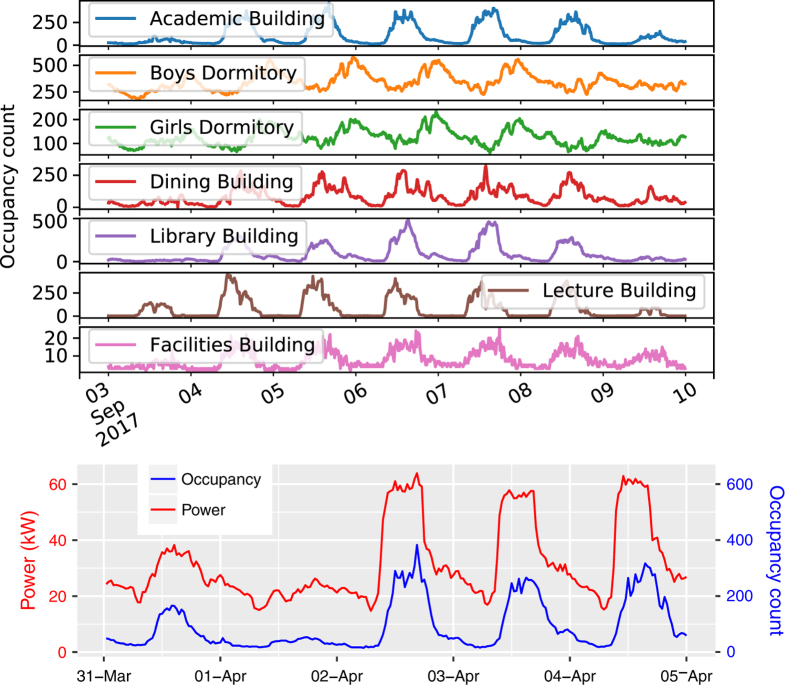
Top: Occupancy of the campus buildings for one week of September 2017. Bottom: Half-hourly power consumption and occupancy of academic building from April 1, 2017, until April 5, 2017.

Figure 7 (top) shows the occupancy pattern of all seven buildings on campus for a week. The occupancy count hardly ever reaches zero as the campus is residential and students work during days as well as nights. Lecture rooms remain closed during night hours, so occupancy reaches to zero in the lecture building at night. Figure 7 (bottom) shows the power consumption and occupancy count of the academic building for five consecutive days at half-hourly intervals. April 1 was a Saturday, so only non-teaching staff and some Ph.D. students were present in the building as compared to April 3–5, which were working days. The Pearson correlation coefficient between power consumption and the occupancy count of the plotted data is 0.89. For all the buildings on campus, we computed the correlation coefficient between the power consumption and the occupancy count using one week (June 11 to June 16, 2017) of data. We chose this duration because there were no missing values in any of the buildings’ data. The correlation coefficient for the academic building, the boys’ dormitory, the dining building, the library, the lecture, and facilities buildings is 0.87, 0.75, 0.75, 0.83, 0.80, 0.71, and 0.87, respectively. A significant correlation (≥0.70) between the power consumption and the occupancy suggests that occupancy data can be used as a parameter for predicting the energy consumption of a building.

Occupancy estimated via SNMP traps have two error sources: (i) when an occupant has more than one device connected to the Wi-Fi simultaneously, and (ii) when an occupant does not connect to Wi-Fi. The former case results in occupancy over-estimation, while the latter case results in occupancy under-estimation. We calculated the occupancy over-estimation, but it is difficult to find the under-estimation due to the unavailability of the required data. However, under or over-estimation may vary across the sessions (mentioned in Table 3), but it remains constant across the days of the same session as the number of students staying on campus remains constant throughout a session. Occupancy over or under-estimation should not limit the study of occupancy data on energy consumption since we found a significant impact of the change in occupancy on the energy consumption duration during our analysis.

**Figure 8:**
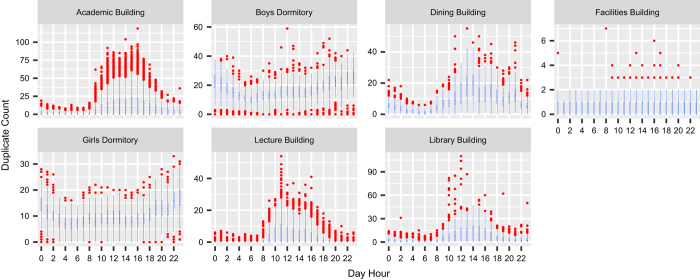
Box plots showing the distribution of occupants (duplicate count) using multiple devices at the same time at different day hours over two months (Aug. and Sept. 2017).

The IT department of the institute maintains a registration directory which lists each current user and the set of devices registered by the user. While knowing MAC addresses from this directory, we counted the number of active devices from SNMP logs pointing to the same user at a specific timestamp as both the registry and SNMP logs contain device MAC addresses. In this way, we calculate the number of duplicate connections for all buildings on the campus. Box plots in Fig. 8 show the distribution of such duplicate connections hour-wise for August and September 2017. The figure shows that the number of duplicates increase during day hours in the academic, the dining, the lecture, and the library buildings, while in the dormitories, it increase during night hours, and in the facilities building, no significant change is found. Box plots show that in the academic building, which witnesses high movement, occupancy count from SNMP overestimates by 50 and in remaining buildings, it overestimates up to 20. We could not estimate the duplicate connections for all the months of the dataset due to the unavailability of the required data as the registration directory is updated in July every year with the new admissions data. During the update, new MAC addresses are added, and older ones are removed.

## Usage Notes

Datasets released in this paper are time-series datasets. Therefore, such data can be analyzed with any software package for time series data. We encourage users to use either R or Python (Pandas) due to their vast collection of open-source data analysis libraries. We also provide an R script on GitHub^28^ with different functions for preliminary data visualization.

In the script, function visualize_data() plots any of the features of an input CSV file. While exploring the dataset, it is observed that it takes time to load high-frequency energy data for plotting, so another function, visualize_data_at_lower_frequency() is used to plot the low-frequency data of the same dataset. Users may use function resample_data_minutely() to downsample the data at a required frequency. Further, for any questions, we encourage dataset users to raise an issue at our GitHub page^29^.

## Additional information

**How to cite this article**: Rashid, H. *et al*. *I-BLEND*, a campus scale commercial and residential buildings electrical energy dataset. *Sci.Data*. 6:190015 https://doi.org/10.1038/sdata.2019.15 (2019).

**Publisher’s note**: Springer Nature remains neutral with regard to jurisdictional claims in published maps and institutional affiliations.

## Supplementary Material



## Figures and Tables

**Table 1:** Details of publicly available electrical energy datasets.

Dataset	Para-meters	Duration	Sampling rate (sec.)	Houses (#)	Data type
AMPds^30^	V, I, f, PF, P, Q, S	2 years	60	1	Agg. & App.
Dataport^31^	P	since 2011	60	>500	Agg. & App.
DRED^32^	P	5 months	1	1	Agg. & App.
ECO^33^	V, I, *ϕ*	8 months	1	6	Agg. & App.
GREEND^34^	P	1 year	1	9	Agg. & App.
REDD^35^	V, P	19 days	15 kHz (Agg.), 3 (Cir.)	6	Agg. & Cir.
Smart*^36^	P, S	3 months	1	3	Agg. & Cir.
UK-DALE^37^	V, I, P	4 years	16 kHz (V, I), 6 (P)	5	Agg. & App.
REFIT^38^	P	2 years	8	20	Agg. & App.
Acronyms: V→ Voltage, I→ Current, f→ Frequency, PF→ Power factor, P→ True power, Q→ Reactive power, S→ Apparent power, *ϕ*→ Phase angle, E→ Energy, Agg.→ Aggregate, App.→ Appliances, Cir.→ Circuits.					

**Table 2:** Building details. Facilities building do not have a centralized HVAC.

Building	Floors (#)	Total area (*ft*^2^)	HVAC area (*ft*^2^)
Academics	5	61308	22785
Lecture	3	17548	12101
Library	4	26401	18354
Dining	4	50191	11840
Facilities	3	09769	NA
Boys Dormitory	6	72745	30120
Girls Dormitory	4	38126	12818

**Table 3:** Occupancy count of boys’ and girls’ dormitories during three sessions for three different years.

Year	Jan.–April		May–July		Aug.–Nov.	
	Boys	Girls	Boys	Girls	Boys	Girls
**2015**	394	182	90	56	389	183
**2016**	428	192	247	144	456	195
**2017**	438	190	254	129	450	205

**Table 4:** Day and night temperature features - mean and standard deviation at IGIA airport and IIIT-Delhi campus over a duration of four months from March to June 2018.

Time	Mean temperature		Standard deviation	
	Airport	IIITD	Airport	IIITD
Day	34.53	34.01	5.64	4.2
Night	28.63	30.44	5.61	4.2

## Steps to compute occupancy count of each building

**Input:** *X* - SNMP traps log for a time period *T*.

**Output:** Time series occupancy count $B_{i}^{o}$ for each of seven buildings for time period *T*, where *i*∊{1,…,7}

**1** Separate *X* into different buildings *B*_*i*_ where *i*∊{1,,7} and represents building number. Each trap in *X* contains a building identifier.

**2 for** ***i*****←1 to 7 do**

**3** *C* ← Extract only ciscoLwappDot11ClientMovedToRunState traps from *B*_*i*_.

**4** **foreach**
trap
*c* in *C* **do** Create a time series ON sequence with start and stop timings of *c*

**5** Bind all ON sequences along the time series index to create a dataframe DF

**6** Perform row wise addition of DF to create final time series occupancy count $B_{i}^{o}$ of the building *B*_*i*_

**7** return $B_{i}^{o}$

**8 end**
